# Electrospun Nanofibers Applied to Dye Solar Sensitive Cells: A Review

**DOI:** 10.3390/ma12193190

**Published:** 2019-09-29

**Authors:** Jesse Gerardo López-Covarrubias, Laura Soto-Muñoz, Ana Leticia Iglesias, Luis Jesús Villarreal-Gómez

**Affiliations:** 1 Facultad de Ciencias Químicas e Ingeniería, Universidad Autónoma de Baja California, Tijuana 22390, Baja California, Mexico; lopez.jesse@uabc.edu.mx (J.G.L.-C.); soto.laura@uabc.edu.mx (L.S.-M.); 2 Escuela de Ciencias de la Ingeniería y Tecnología, Universidad Autónoma de Baja California, unidad Valle de las Palmas, Tijuana 21500, Baja California, Mexico

**Keywords:** electrospun nanofibers, electrospinning, electrolyte, solar cells, DSSC, photoanode, counter electrode

## Abstract

In recent decades, there has been an increase in the research for the development and improvement of dye sensitized solar cells (DSSCs), owing to their singular advantages such as greater efficiency in energy conversion and overall performance in adverse environmental conditions. Therefore, work is carried out to enhance the energy efficiency of the components of the DSSCs: photoanode, counter-electrode, electrolyte, and dye sensitizer layer. Electrospun nanofibers in particular, have showed to be a novel alternative as components in DSSCs, mainly for energy conversion and as collector materials due in part to their tridimensional structure, high contact surface area and conductivity. Moreover, the incorporation of metallic compounds into nanofibers is advantageously employed in the electrospinning technique, owing to their conductivity and optical properties. Therefore, the present work consists of a detailed recompilation of the use of electrospun nanofibers loaded with metallic compounds and their application in DSSCs. The functionality of the components of DSSCs, parameters and experimental conditions of electrospinning, such as the intrinsic aspects in the polymer solution, are discussed and applied to the photoanode, counter-electrode and electrolyte of the DSSC. Lastly, the use of the electrospinning technique in combination with the use of metallic compounds could provide a great approach for the developing of DSSCs, with superior efficiency, high stability and durability.

## 1. Introduction

For the last 20 years, advancements in the generation of energy through solar cells has been remarkable, not only due to increased energy efficiency, but also because of improvements which can be seen in its design and manufacturing [1]. Currently, there are three generations of solar cells: (a) silicon wafers comprised the first generation; (b) thin-film solar cells make up the second generation, and finally, (c) organic solar cells encompass the third generation of solar cells [2]. Amongst these, silicon solar cells have the highest energy conversion with efficiencies up to 26% [3]. Dye sensitized solar cells (DSSC) which are included in the third generation, have the greatest efficiency of all other types of organic cells, with an energy conversion of up to 10% [4]. In addition to their more effective ability to capture solar diffuse radiation, they have better performance in low light environments such as indoor environments and on cloudy days [5].

An advantage in the design of DSSCs is the use of flexible and lighter materials, which consequently increases the number of applications in which the DSSCs can be used. Furthermore, DSSCs bear certain advantages compared to the first and second generation solar cells: a lower manufacturing cost in conjunction with a market low cost of the components, makes the cost of a DSSC about 10–20% of the manufacturing cost of silicon cells [6]. The increase in the energy conversion efficiency is a great challenge to overcome in the market entry of DSSCs. To achieve this objective, modifications have been made to the components of the DSSC, as well as to their manufacturing processes [7]. The electrospinning technique is an option to increase the efficiency of DSSCs, because the resulting nanofibers increase the contact area, garnering a greater absorption of solar energy [8]. In this work, a summary of the components of DSSCs is made, as well as its operation. A brief review of the electrospinning technique and its use in the manufacture of DSSC is discussed, presenting an updated comparison of electrospun compounds and their efficiency; and thus providing the basis for the development in the manufacture and design of DSSCs.

## 2. Dye Solar Sensitive Cells

### 2.1. Background

The first DSSC solar cells date back to 1972, when cells were prepared using chlorophyll as a sensitizing dye and zinc oxide as a photoanode, however, efficiency was very low (2.4%) due to its low surface area [9,10]. In 1991, Michael Grätzel and Brian O’Regan developed a solar cell using a combination of layers; a nano-structured titanium (IV) oxide (TiO_2_) layer with a Ruthenium (II) complex as a photoanode, and with this change in the manufacturing process an increase to 7% in the efficiency of the DSSC was achieved [11].

### 2.2. Components of a DSSC

**Figure 1:**
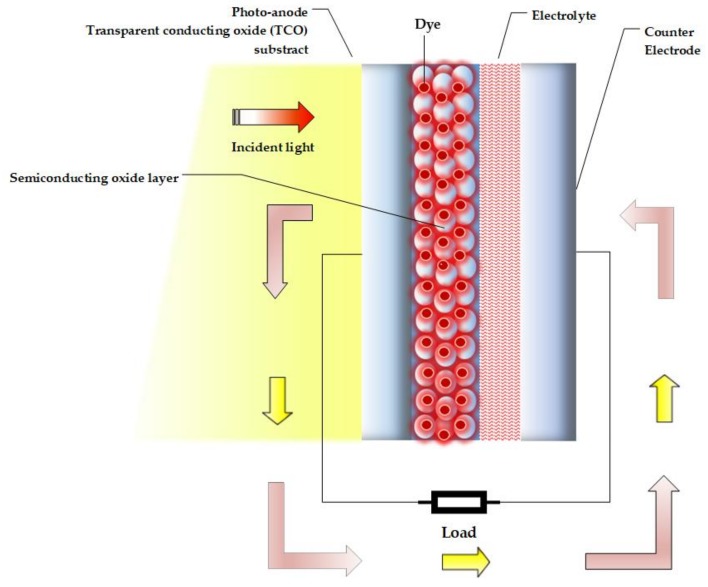
Diagram of the structure of a DSSC (based on [13]).

The structure of the DSSC is illustrated in Figure 1. The main components are: photoanode, dye sensitizer layer, electrolyte and counter-electrode [5,10,12,13]:

#### 2.2.1. Photoanode

The photoanode consists of a metal oxide semiconductor deposited on the surface of a transparent conductive oxide substrate (TCO). The TCO allows the collection and transfer of electrons to an external circuit. Usually, a fluoride doped tin oxide (FTO) is used as the TCO; this is due to its excellent electrical conductivity (5.8 × 10^−2^ S·cm^−1^), optical transparency and 75% of transmittance approximately [10,13,14]. Titanium (IV) oxide compound (TiO_2_) is the most common semiconductor used in depositions in the TCO of photoanodes for DSSC [5,9,10,11].

#### 2.2.2. Dye Sensitizer Layer

The dye sensitizer in the DSSC is responsible for absorbing the light generated from the electron current through photo excitation and is thus affixed to the photoanode [15].

#### 2.2.3. Electrolyte

The redox electrolyte in the DSSC works as a transport path to transfer electrons from the counter-electrode to the oxidized dye, thus compensating for the loss of electrons in the dye. The electrolyte in the DSSC can be classified as a: liquid, quasi-solid and solid. The most common electrolyte used in DSSC are in the liquid state; usually a iodide/triiodide based compounds [12,16,17].

#### 2.2.4. Counter-Electrode

The counter electrode is responsible for taking the electrons from an external circuit and dispersing them in the electrolyte [17]. Consequently, it is crucial that the counter electrode has a good reduction reaction rate, as well as a high electrical conductivity and catalytic activity. Platinum (Pt) is used as a standard catalyst in DSSC, because of its high current density and catalytic efficiency. However, the use of Pt elevates the cost of the device [10]. Accordingly, electrospun nanofibers with metallic oxides can be used as replacement for Pt as counter electrodes due to their tridimensional structure and high surface area. These 3D structures are also called scaffolds and have better light-absorbing capacity due to their larger surface area compared to 1D structures. In the electrospinning technique, a polymeric solution drop is attracted by an electrical field creating nanofibers, which fall into a static collector. Fibers are then randomly deposited one over another, constructing a porous scaffold and creating a vast surface area. Hence, the more surface area that is present, the more electrons can be displace at the same time to the electrolyte compartment [10,17].

### 2.3. Working Principle

The operating principle of a DSSC can be understood by the following [17,18,19,20,21,22,23]: (a) light comes in contact with the dye, (b) photons of light are trapped by the dye, which causes an excitation of electrons, and the electrons are transmitted to the conduction band of the semiconductor (photoanode), (c) the photoanode collects the electrons and transports them to an external circuit, which transmits them to the counter-electrode, (d) the counter-electrode transfers electrons to the electrolyte, which serves as an intermediate between the counter-electrode and photoanode, and, (e) the electrolyte restores the lost electrons to the photoanode.

### 2.4. Applications

DSSCs have made their way into the world of solar cells, due to their advantages over first- and second-generation solar cells; not only for their low cost, but for operating in non-standardized conditions, making them ideal for places where the environmental conditions vary greatly. DSSC for example, are efficient in low light environments, e.g., at dusk, on cloudy days or even indoors. Therefore, DSSC can be applied to many applications in areas such as panels, tiles, facades, solar roofs and smart sensors in automotive and nautical industries, among many others [24].

## 3. Electrospinning Technique

**Figure 2:**
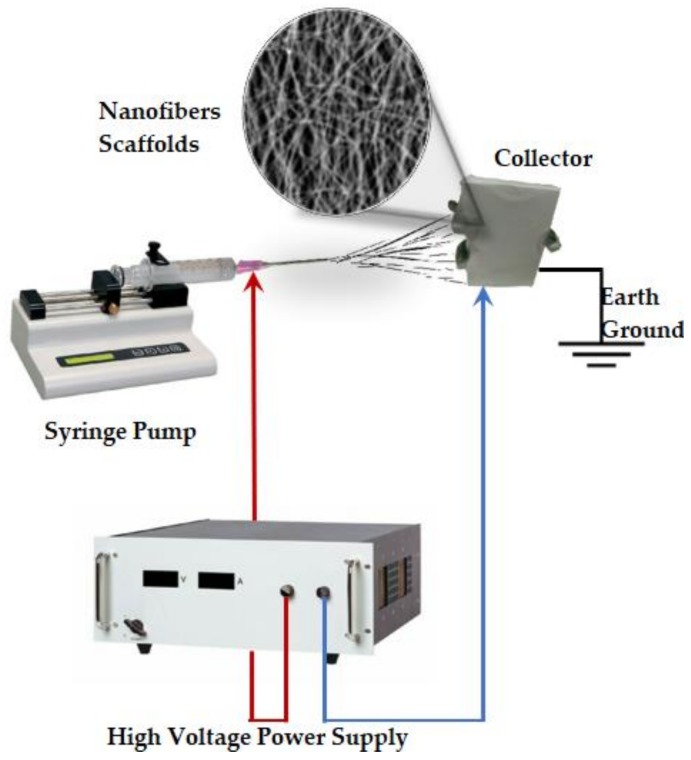
Electrospinning device set up (based on [26]).

Electrospinning is a technique used for the formation of fibers and manufacture of fibrous scaffolds; its applications have increased substantially in recent years; particularly in the fields of medicine and engineering (R&D). The electrospinning technology is based on the injection of a conductive polymer solution, electrically charged and directed towards a collector, against an electrode that is grounded closing the circuit. The solution then passes through a field of electrostatic forces ranging from 20 to 50 kV, which produces a stretching effect that forms fibers from one nanometer to one micrometer (Figure 2). “Upon effect of electrostatic forces, the polymeric solution is attracted towards the opposite electrode and a straight jet of solution is formed from a conical protrusion, often called a Taylor cone, leading to fibers with diameter in submicron range” [25], the solvent then evaporates and is deposited in the collector.

### 3.1. Parameters and Conditions of Electrospinning

The parameters to mainly consider are: applied voltage, flow velocity, type of collector, and distance between the collector and the tip of the dispenser of the polymeric solution. The experimental conditions are ideally carried out in a controlled atmosphere (pressure, temperature and low humidity) in the electrospinning chamber, as well as, “air velocity inside the chamber” [26]. An important and intrinsic parameter to consider in the preparation of the solution that will be independent of the compound to be chosen, is the molecular weight of the polymer, since the optimum concentration of the polymer and the final viscosity of the solution obtained will depend on it.

### 3.2. Polymeric Solution Used to Produce the Photovoltaic Effect

In principle, the selection of the polymer should be made according to the characteristics that match according to the application. In the case of the photovoltaic industry, important parameters such as the free flow of electrons, characteristic of the conductivity of the material and mobility between empty spaces, are some aspects that should be considered in the synthesis of solar cells. Generally, the conductivity mechanism in conducting polymers is based on the motion of charged defects within the conjugated framework. These defects can be positive (p-type) or negative (n-type) and result from the oxidation or reduction of the polymer, respectively [27].

### 3.3. Fiber Morphology Characteristics

Particle or suspension electrospinning refers to dispersing particles in the polymer solution and electrospinning the mixture. Various nanoparticles have been electrospun at low loadings including magnetite, TiO_2_, CaCO_3_, fumed silica, carbon black, iron and nickel, with an average particle diameters of less than 100 nm and fiber size on the order of 200–2000 nm [28].

## 4. Electrospinning Applied to DSSC

Despite the multiple advantages presented by the DSSC, there is a problem with their low efficiency; consequently, in recent years there have been many studies where strategies are proposed to increase their efficiency. One way to achieve this goal is to develop electrospun components for the DSSC, such as photoanodes, counter-electrodes and electrolytes [29].

### 4.1. Photoanodes

**Figure 3:**
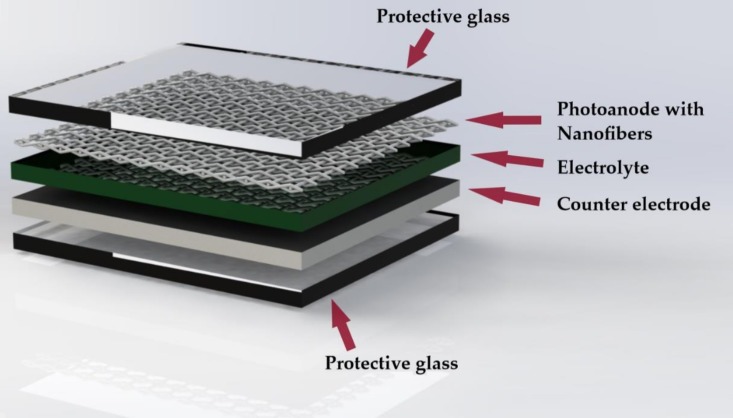
Photoanode with electrospun nanofibers in a DSSC.

In the photoanode of a DSSC, morphology and structure play a very important role in the performance of electron transport [29]. Photoanodes composed of semiconductor nanoparticles are commonly used as they provide active spaces for dye adsorption, but its grain boundaries may cause an unexpected detachment that brings about a low electron transport and efficiency. Several studies have been carried out for the development of photoanodes with materials with one-dimensional morphology (1D). Therefore, cellulose acetate nanotubes and nanofibers compounds are a very promising option for its use in DSSC (Figure 3) [8].

TiO_2_ is the most widely used semiconductor, however, compounds such as Sn (IV), Zn (II), Ni (II), have also been studied for the manufacture of electrospun nanofibers used as photoanode [30,31,32,33,34,35,36,37,38,39,40]. Table 1 summarizes the most significant results concerning the efficiency of the above discussed nanofibers as photoanode. The nanofibers discussed in this review are arranged in Table 1 in order of ascending efficiency.

TiO_2_ nanofibers are not the only scaffolds used as photoanode. ZnO/poly (vinyl acetate), composite nanofiber mats (Table 1, entry 1), were directly electrospun onto a glass substrate coated with F: SnO_2_ for its application in DSSCs. After being electrospun, these fibers were hot pressed at 120 °C and calcined at 450 °C. This resulted in a multiple nanofiber network composed of a twisted structure of 200–500 nm diameter cores with ~30 nm single grains. The DSSCs using ZnO nanofiber mats exhibited a conversion efficiency of 1.34% under 100 mW∕cm^2^ (AM-1.5G) illumination [31].

Currently, there is great interest in the manufacture of vertically aligned nanowires from electrospun nanofibers, with the objective of having an efficient electron transfer. Krishnamoorthy et al. (2011) reported for the first time the fabrication of vertical nanowires of TiO_2_ from electrospun nanofibers (Table 1, entry 2). This study resulted in an energy conversion efficiency of 2.87%. Authors sustain that this approach can be a better alternative to the currently available methods, such as hydrothermal synthesis and template assisted fabrication, because the diameter, height, and spacing between the wires can be effectively controlled by this electrospinning [32]. Moreover, nanowires of Nb_2_O_5_ were developed in three polymorphic forms (Table 1, entry 3), pseudo-hexagonal, orthorhombic, and monoclinic, by the electrospinning technique followed by a subsequent annealing. The pseudo-hexagonal phase, showed higher device performance owing to its higher specific surface area compared with the other two. However, the monoclinic phase gave superior performance with respect to dye-loading. Studies on the charge transport properties of the nanofibers, using electrochemical impedance spectroscopy and open circuit voltage decay, indicates that the monoclinic phase has high resistance against charge recombination and improved electron lifetime, compared with the other phases and conventional TiO_2_ nanostructures. The monoclinic Nb_2_O_5_ is likely to be a material of choice as a photoelectrode in dye-sensitized solar cells, if mesoporous particles with large surface area could be synthesized [33].

Graphene oxide (GO)-incorporated into TiO_2_ nanofibers (Table 1, entry 4), have also been proposed as a working electrode for dye sensitized solar cells (DSSCs). The presence of graphene oxide increases the amount of dye absorption, which leads to high migration of photoinduced electrons to the conduction band of the collection electrode, inhibiting electron recombination. Additionally, the presence of GO improves the electron transport from the films to the fluorine doped tin oxide (FTO) substrates. Accordingly, a remarkable enhancement in power conversion efficiency of 4.52% was achieved, by utilizing 0.5 wt% GO-incorporated TiO_2_ nanofibers as working electrode, which is higher than the conversion efficiency in case of pristine TiO_2_ nanofibers (1.54%). A higher amount of graphene oxide, results in a decrease in the power conversion efficiency [34].

Li et al. (2014) [35], also used electrospun TiO_2_ nanofibers to enhance the conversion efficiency of ZnO-based DSSC. According to the authors, the system TiO_2_/ZnO (Table 1, entry 5) composite photoanode, provides a direct transport pathway for electron injection to increase electron transfer efficiency. The results indicated that the light scattering of the photoanode film was increased and that electron recombination was suppressed when the appropriate amount of hollow TiO_2_ nanofibers were added to ZnO. The maximal energy conversion efficiency reached 4.59% by adding 10 wt% of hollow TiO_2_ nanofibers, which is 62% higher than that of DSSC based on pure ZnO nanoparticles (~2.84% PCE).

Gao et al. (2012) [36], reported a high-efficiency photoelectrode for dye-sensitized solar cells (DSSCs), and some advantageous features, such as fast electron transport, slow interfacial electron recombination and large specific surface area, were discussed. Nevertheless, these properties cannot be simultaneously present. The power conversion efficiency (PCE) of the SnO_2_ nanofibers-TiO_2_ (SnO_2_ NF-TiO_2_) was ~4.61% (Table 1, entry 6); a similar value compared to the reported ~4.82% for TiO_2_ nanoparticles films in (P25 film) DSSCs.

Novel TiO_2_/Nb_2_O_5_ core–sheath nanofibers (NFs) (Table 1, entry 7) were prepared via co-electrospinning. The TiO_2_/Nb_2_O_5_-based DSSCs were superior to just TiO_2_-based nanofibers. The conversion efficiency was enhanced from 4.5 to 5.8%, which corresponds to an increase of ca. 30% [37].

TiO_2_ nanofibers that have been doped with silver (Table 1, entry 8), increased the amount of dye loading, causing a higher short-circuit current in the photoanodes. This strategy was used to increment the open circuit voltage. This process enhanced the exit of dye molecules, which were rapidly split into electrons; so DSSCs with these nanofibers stopped the recombination of the electronic process. The conversion efficiency of TiO_2_ photoelectrode-based DSSCs was 4.74%; which increased to 6.13% after adding 5 wt% of Ag-doped TiO_2_ nanofiber (ATN) into TiO_2_ films. The electron lifetime of DSSCs with ATN increased from 0.29 to 0.34 s and the electron recombination was reduced [38].

ZrO_2_ electrospun fibers (Table 1, entry 9) were also reported for the application in dye-sensitized solar cells (DSSCs). The addition of ZrO_2_ fibers into TiO_2_ electrodes provided good dye loading and an effective electron pathway, increasing the charge transfer in the TiO_2_/electrolyte interface over conventional TiO_2_ electrode. Energy conversion efficiency of DSSCs with a TiO_2_–ZrO_2_-700 composite electrode was 6.2%, an increase of 26.5% compared to 4.9% of pure TiO_2_ electrode. The electron recombination times of pure TiO_2_ and TiO_2_–ZrO_2_-700 electrode were 15.94 and 21.69 ms, respectively [39]. The data obtained by the TiO_2_-ZrO_2_ nanofibers surpass the given results of a study conducted by X. Luan and Y. Wang in 2014, where a thin film made of TiO_2_-ZrO_2_ achieved a 6.12% energy conversion efficiency [36].

Finally, the most efficient nanofibrous system is fabricated with TiO2-Graphene nanofibers (Table 1, entry 10). These fibers reported a minimal presence of graphene between 0.0–0.7 wt%. TiO_2_-graphene composites fibers presented a two-fold higher efficiency than that of the electrospun TiO_2_ fibers, obtaining up to 7.6% [40], which is the best reported efficiency of all nanofibers compared. Furthermore, these values are higher than the results given by a non-electrospun layer of TiO_2_-graphene (in 2011) with a reported efficiency of 6.86% [37].

### 4.2. Counter Electrode

In Table 2, a comparison of efficiencies of different electrospun polymeric composite nanofibers, proposed as a counter electrode is presented, as before they are arranged in ascending order of efficiency [41,42,43,44,45,46,47,48,49,50,51]. The counter electrode (CE) plays a key role in dye-sensitized solar cells (DSSCs), because it mediates and facilitates the electron transfer from the external circuit to the redox couple, to complete the DSSC circuit [41].

**Figure 4:**
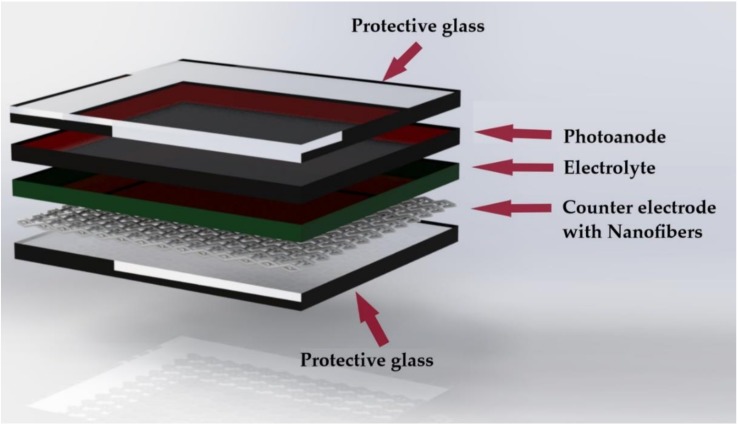
Counter electrode with electrospun nanofibers in a DSSC.

Pt-based counter electrodes are most often used in the DSSC for their excellent performance. Their manufacture is based on thermal deposition and vacuum bombardment; which requires high temperatures and complex installations [41]. Therefore, alternatives to these high operating costs are needed, in addition to the cost of Pt (II) compounds. Experiments with carbonaceous materials have been carried out in addition to electrospun conductive polymers for use as counter electrodes in the DSSC (Table 2, Figure 4) [8,41].

Cobalt-titanium carbide nanoparticles (Co-TiC NPs) embedded on electrospun carbon nanofibers were studied (Table 2, entry 1), the results showed that the introduced composite (metal carbide) could enhance both methanol electro-oxidation and electrochemical stability (189 mV and ∼90 mA·cm^−2^, respectively), as well as the low onset potential and high current density of the composite electrode. Moreover, these fibers were effective, providing stable electro catalytic activity (ECA) and conductivity, indicating that the composite can improve catalytic activity in triiodide reduction [42].

In the same way, kesterite Cu_2_ZnSnS_4_ (CZTS) nanofibers were fabricated by electrospinning process (Table 2, entry 2). These CZTS nanofibers showed optical properties with a strong absorption in 300–550 nm range with band gap energy of 1.5 eV [43]. Another research group replaced Pt with titanium carbide (TiC) embedded with electrospun carbon nanofibers (CNFs) (Table 2, entry 3). The photovoltaic performance demonstrated that the DSSC fabricated using 10 wt% TiC embedded CNFs as CE had very close photo energy conversion efficiency (PCE) than the conventional Pt. This is attributed to the synergistic effect of TiC with the larger electrocatalytic surface area of CNFs, which plays a substantial part in the improvement of photovoltaic performance of DSSC [44].

Rameez et. al. (2016) [45], reported bimetallic (Ni–Co) nanoparticles-incorporated to electrospun carbon nanofibers (CNFs) as a counter electrode (Table 2, entry 4). In this study, the PCE of DSSC was nearer to the power conversion energy of the counter electrode assembled with standard Pt. Hence, authors proposed that Ni–Co nanoparticles-incorporated CNFs can be applied as a cost-effective alternative counter electrode for DSSCs.

In 2015, Saranya et al. [46], studied earth-abundant bimetallic (Fe–Ni) nanoparticle-embedded carbon nanofibers (CNFs) for its potential use as counter electrodes (Table 2, entry 5). These electrospun fibers had power conversion efficiency (PCE), almost comparable to the same fabricated using standard platinum; owing to the CNFs’ large surface area and random orientation; as well as an interconnected porous morphology with graphitized structure that also enhanced the contact with a large quantity of the ionic liquid electrolyte.

Rare earth elements have also been used for the fabrication of electrospun nanofibers used as CE, Ru(III) nanofibers consisting of nano-sized grains are used instead of the typical Pt counter electrode for DSSCs (Table 2, entry 6). These nanofibers were fabricated by electrospinning followed by post-calcination and hydrogen reduction. The resultant Ru nanofibers exhibit properties of improved photovoltaic performance, such as, lower charge transfer resistance (12.5 Ω·cm^−2^), higher short-circuit current density (14.77 mA·cm^−2^), and higher PCE (6.23%) which is comparable to a commercial Pt counter electrode. The improved photovoltaic performance of the counter electrode in the DSSC was attributed to the combined effects of small grain size, which resulted in a high number of electrochemical sites, a high electrical conductivity that lead to improved electro catalytic activity, and a unique network structure that allowed for rapid electron transfer and rapid diffusion of the electrolyte [47]. The efficiency obtained by the Ru(II) nanofiber is greater than the Ru film studied in 2012 by J. Han et al., as this film showed an efficiency of 3.40% [39].

Nickel cobalt sulfide (NiCo_2_S_4_, NCS) particles were prepared through sulfurization of NiCo_2_O_4_ (NCO) electrospun nanofibers (Table 2, entry 7). The bimetallic sulfide was used as CE for DSSC and exhibited an excellent PCE of 7.12%, which is higher than the dye-sensitized solar cells using NCO (5.24%) and Pt CEs (7.05%). Systematic electrochemical characterization suggests that this outstanding performance of NCS might be related to the improved electro catalytic ability and electrical conductivity. Authors agreed, that due to the low-cost synthesis and outstanding electrochemical performance, the NCS counter electrode have great potential for applications in dye-sensitized solar cells [48].

Another example of the use of rare earth elements in DSSC manufacturing is the porous graphene-doped copper indium disulfide/carbon (p-GN@CuInS_2_/C) composite nanofibers (Table 2, entry 8). A dye-sensitized solar cell assembled using the p-GN@CuInS_2_/C nanofibers as the counter electrode (CE) delivered a photoelectric conversion efficiency of 7.23%, which was higher than the efficiencies of DSSCs assembled, using the samples without graphene (6.48%) and with the CuInS_2_/C nanofibers (5.45%). It was also much higher than that of the DSSC with a Pt CE (6.34%). The excellent photoelectric performance of the p-GN@CuInS_2_/C CE was attributed to its special hierarchical porous structure, which facilitated permeation of the liquid electrolytes and provided additional active catalytic sites for the oxidation reaction of the electrolytic (I^−^/I^3−^). The doping of reduced graphene oxide (RGO) resulted in the well-dispersed growth of CuInS_2_ nanocrystals in the carbon nanofibers, which further increased the number of active catalytic sites and promoted electron and ion transfer [49].

Aboagye et al. (2015) [50], (Table 2, entry 9) reported a transparent fluoride-doped tin oxide (FTO) conductive glass with a thin layer coating of platinum (Pt), which was studied as a counter electrode. The widespread use of Pt as counter electrode in DSSCs is due to its catalytic capability for I_3_^−^ reduction in the electrolyte. Pt can be affected by the corrosive nature of I^−^/I_3_^−^ redox couple, which makes it a less desirable candidate for use in industrial scale manufacturing. To improve the system, carbon nanofibers with surface-attached Pt nanoparticles were prepared by stabilization and carbonization of electrospun polyacrylonitrile (PAN) nanofibers (Table 2, entry 9). Compared to conventional counter electrode, carbon-Pt CE exhibited larger open circuit voltage (Voc), moreover, the electrospun DSSCs demonstrated excellent solar energy conversion efficiencies in the range of 7% to 8%.

Finally, a nickel incorporated carbon nanotube/nanofiber composite (Ni-CNT-CNF) was used as a low-cost alternative to Pt as counter electrode (CE) for dye-sensitized solar cells (DSSCs) (Table 2, entry 10). Measurements based on electrochemical impedance spectroscopy (EIS) showed that the charge transfer resistance (R_ct_) of the Ni-CNT-CNF composite electrode was 0.71 Ω·cm^2^, much lower than that of the Pt electrode (1.81 Ω·cm^2^). Such a low value of R_ct_ indicated that the Ni-CNT-CNF composite carried a higher catalytic activity than the traditional Pt CE. By mixing with CNTs and Ni nanoparticles, the series resistance (R_s_) of the Ni-CNT-CNF electrode was measured at 5.96 Ω·cm^2^, which was close to the R_s_ of 5.77 Ω·cm^2^ of the Pt electrode. The DSSCs based on the Ni-CNT-CNF composite CEs yielded an efficiency of 7.96% with a short circuit current density (J_sc_) of 15.83 mA·cm^−2^, open circuit voltage (V_oc_) of 0.80 V, and a fill factor (FF) of 0.63, which was comparable to the device based on Pt, that exhibited an efficiency of 8.32% with J_sc_ of 15.01 mA·cm^−2^, V_oc_ of 0.83, and FF of 0.67 [51].

### 4.3. Electrolyte

Liquid electrolytes are most often employed in DSSC. However, there are drawbacks in their use. The spill of the liquid is especially a major concern, and thus, greater care must be taken in the hermetic seal of the solar cell. An alternative route to this type of electrolyte are solid electrolytes such as polymers, although they have the disadvantage of low efficiency due to lower electron injection [29].

**Figure 5:**
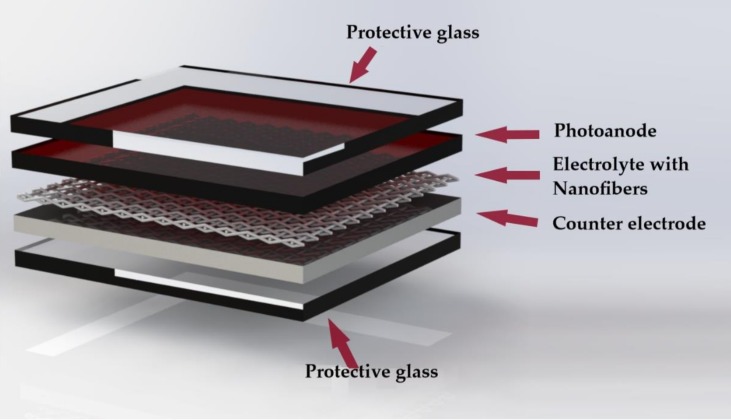
Electrolyte with electrospun nanofibers in a DSSC.

Electrolytes in a quasi-solid state are another alternative for replacement in DSSC. In recent studies, electrospun nanofibers have been used as an alternative for quasi-solid electrolytes (Figure 5). These investigations have yielded results of high efficiencies which can compete with the efficiencies of liquid electrolytes, in addition to being more stable electrolytes, because they avoid several potential problems such as the complicated sealing and non-volatilization of DSSCs; contrary to the liquid component [52].

Table 3 presents a comparison of electrolytes based on electrospun nanofibers, and their composition, experimental conditions for electrospinning and main results are presented. Nanofiber are shown in ascending order of efficiency [53,54,55,56,57,58,59,60,61,62].

In 2014, M. Sethupathy et al. [53], created a DSSCs with a polymeric electrolyte based on a mixture of PVDF [poly (vinylidene fluoride)] and polyacrylonitrile (PAN) (Table 3, entry 1), and although their efficiency was only 3.09%, they achieved an open circuit voltage (Voc) of 0.74 V, similar to the previous reported voltage of electrolytes based on PVDF. In addition, the nanofiber porosity and ionic conductivity was 83.6% and 6.12 × 10^−2^ S·cm^−1^, respectively.

Weerasinghe et al. (2017) [54], elaborated a quasi-solid electrolyte using cellulose acetate (CA) nanofibers (Table 3, entry 2), which showed an efficiency of 4.0%. Moreover, these CA nanofibers are biodegradable; a feature that should be taken into account for future environmental or biomedical applications.

In 2014, electrospun nanofibers of silicon oxide (IV) SiO_2_ (Table 3, entry 3) were used as an electrolyte by X.G. Zhao et al. Although in this case the energy conversion efficiency only reached 4.85%, a high short circuit current of 13.63 mA/cm^2^ was observed, as well as a high interfacial stability between the polymer electrolyte and semiconductor electrodes [55]. As mentioned earlier, electrospun PAN compounds have also been used in electrolytes for DSSC. In 2014, Dissanayake et al. created an electrolyte in a quasi-solid state, using electrospun PAN nanofibers submerged in a solution containing potassium iodide (KI), propylene carbonate (PC) and iodine (I_2_) (Table 3, entry 4), with an energy conversion efficiency of 5.2% [56].

In an effort to improve the performance of DSSCs, electrospun compounds of polyvinylidene fluoride (PVDF) have been used as a quasi-solid electrolyte (Table 3, entry 7). S. Park et al. (2011) [59], reported two variations of nanofibers which were made of PVDF hexafluoropropylene (PVDF-HFP) and PVDF-HFP/polystyrene (PS). The purpose was to compare the effect of each compound in the DSSC. For the both types of nanofibers, different weight ratios were employed. As a conclusion, it was observed that variations in the proportion and composition of nanofibers did not influence ionic conductivity, absorption, porosity and photovoltaic performance. However, PVDF-HFP/PS nanofibers (Table 3, entry 7) showed a better efficiency 5.75%, compared to the compound without polystyrene 5.39%.

The efficiency obtained in PVDF-HFP nanofibers (Table 3, entry 7) by S. Park et al. [59], is similar to the efficiency shown by Bandara et al. (2018) [57] (Table 3, entry 5) of 5.36% using the same compound years later. In this study a crystallinity analysis of the PVDF nanofiber was carried out using diffraction scanning calorimetry (DSC) thermograms, where a 14% decrease in crystallinity was observed compared to PVDF, without being electrospun.

In another study conducted by S.J. Seo et al. (2011) [58], an electrolyte in a quasi-solid state based on bicarbonated poly (phenylene oxide)/poly (acrylated) nanofibers (BPPO) (Table 3, entry 6) was prepared, thus fabricating a non-fluorinated polymer membrane. The objective of using BPPO was to observe the changes in the efficiency of the DSSC due to the increased in Lewis basicity of the BPPO structure. As a result, an energy conversion efficiency of 5.4% was obtained.

V. Murugadoss et al. (2018) [60], improved PAN nanofibers by adding nanoparticles of cobalt sulphide (Table 3, entry 8), with this electrolyte an efficiency of 7.41% was achieved. This is due to the increase in ionic conductivity and large interfacial area for charge transfer at the electrode/electrolyte interface.

M. Fathy et al. (2016) [61], created electrospun membranes for quasi-solid electrolytes based on PVDF with poly (methyl acrylate) (PMMA) and polyethylene glycol (PEG) (Table 3, entry 9). The weight ratio of the PMMA-PVDF and PMMA-PVDF/PEG solutions was compared. The PMMA-PVDF/PEG solution was the most efficient at 7%; additionally, an ionic conductivity of 3.2 × 10^−3^ S·cm^−1^ was obtained. Another factor that was significantly modified with the presence of PEG in the PMMA-PVDF solution was the average fiber diameter; which decreased from 500 nm to 223 nm [61].

Continuing with electrospun compounds based on PVDF, I.A. Sahito et al. (2017) [62], added a lithium chloride salt (LiCl), in order to increase the mobility of ions in the sensitizer dye in DSSC (Table 3, entry 10). The electrolyte composed of PVDF-LiCl yielded a considerable increase in efficiency of the DSSC with a value of 8.73%, which is the highest efficiency reported for an electrolyte based on electrospun nanofibers. In conclusion, PVDF-LiCl nanofibers (Table 3, entry 10) showed the best efficiency of all the nanofibers discussed. Furthermore, the conductivity exhibited by these nanofibers is more relevant in the electrolyte component, e.g., PVDF-PAN showed a conductivity of 6.12 × 10^−2^ S·cm^−1^ whereas PAN nanofibers was only 3.2 × 10^−3^ S·cm^−1^. In comparison, the reported conductivity for a conventional polymeric electrolyte, is 7.37 × 10^−3^ S·cm^−1^ [63]. This data suggests that PVdF-PAN nanofibers showed better conductivity that conventional electrolytes, facilitating the electron transfer trough the DSSC.

**Figure 6:**
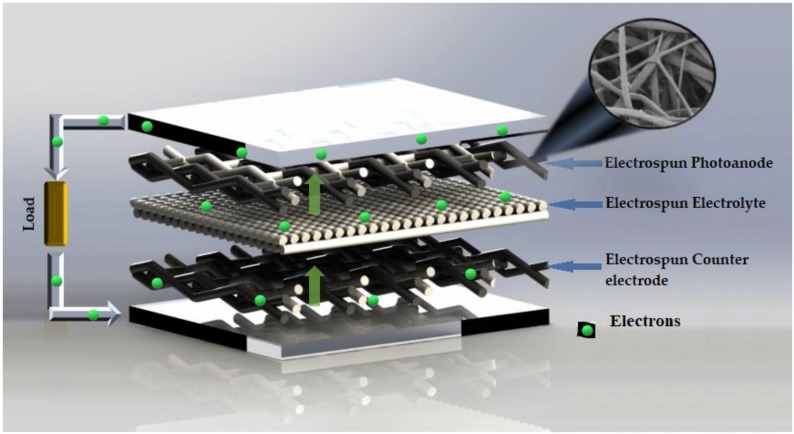
Position of electrospun nanofibers in DSSC (photoanode, electrolyte and counter electrode) (based on [64]).

Figure 6 summarizes the position where electrospun nanofibers can be located to replace some components in DSSC. The flow of electrons as they move through the DSSC can be observed, and this movement is facilitated by the nanofibers.

## 5. Conclusions

In this review, the use of electrospun composite nanofibers as components for dye-sensitized solar cell (DSSC) was discussed. Nanofibers can be applied in three different components in the structure of DSSC: photoanode, counter electrode and electrolyte. In the case of the photoanode component, TiO_2_ nanofibers are the most commonly used, due to their high efficiency, fast electron transport, slow interfacial electron recombination and large specific surface area. Other systems with ZnO have been proposed, but most of the strategies maintain a TiO_2_ base; nonetheless adding graphene to TiO_2_ nanofibers considerably improved the electron transport efficiency, achieving the highest efficiency of all of the fibrous scaffolds discussed. In the case of the counter electrode, the main idea behind the use of nanofibers is the reduction of platinum (Pt) due to its high cost. Researchers are using rare earth elements and graphene oxides as replacements and are obtaining promising results, and Ni-Carbon fibers exhibited the best results. Finally, in the electrolyte component, the idea is to minimize the use of liquid solutions because of its disadvantages such as spills and delicate handling, PVDF-LiCl nanofibers presented the best results for its use in this DSSC component. Furthermore, electrospinning is a versatile, reproducible and low-cost technique which is commonly used in various applications. Electrospun nanofibers offers several advantages for the fabrication of DSSC, such as high surface area, a tridimensional structure, stability, and adequate mechanical properties. Hence, these fibers are particularly convenient in the manufacture of flexible DSSC, moreover, fibers occupy a smaller space in the components, so final DSSC can be fabricated with a tiny thickness. Still many challenges need to be overcome to completely replace the standards components in DSSC with electrospun nanofibers, such as the reproducible concentration of metals over the polymeric nanofibers, equal or higher efficiencies compared to the conventional DSSC, as well as the high cost of some polymers and solvents used in the electrospinning technique.

## Figures and Tables

**Table 1:** Comparison of the characteristics of electrospun nanofibers for photoanodes.

Entry	Composition	Experiment Conditions	Voc ^1^ (V)	Jsc ^2^ (mA/cm^2^)	FF ^3^	η ^4^ (%)	Ref.
1	ZnO	Solution: PVA, DMF, Zn(CH_3_COO)_2_, HAc. * Voltage: 15 kV Rate: 0.015 mL/h	0.60	3.58	0.62	1.34	[31]
2	TiO_2_	Solution: PVP, Ti(Iso), HAc, EtOH. Distance: 20 cm Voltage: 15 kV Rate: 0.2 mL/h	0.782	5.71	0.64	2.87	[32]
3	Nb_2_O_5_	Solution: PVP, NbEt, EtOH, HAc. Rate: 2 mL/h	0.77	6.68	0.59	3.05	[33]
4	TiO_2_-GO	Solution: Ti(Iso), PVA, HAc, GO. * Voltage: 16 kV	0.784	9.41	0.61	4.52	[34]
5	TiO_2_-ZnO	Solution: TBT, EtOH, PVP. * Rate: 0.8 mL/h	0.59	13.15	0.58	4.59	[35]
6	SnO_2_-TiO_2_	Solution: SnCl_2_·2H_2_O, EtOH, DMF, PVP. * Voltage: 13.5 kV	0.723	14.71	0.48	4.61	[36]
7	TiO_2_-Nb_2_O_5_	Solution: Ti(Iso), PVA, HAc, DMF, EtOH, C_10_H_25_NbO_5_. * Voltage: 15 kV Rate: 0.5 mL/h	0.79	11.6	0.63	5.8	[37]
8	Ag- TiO_2_	Solution: Ti(Iso), PVP, HAc, EtOH, AgNO_3_. * Voltage: 20 kV Rate: 0.05 mL/h	0.68	14.93	0.60	6.13	[38]
9	TiO_2_-ZrO_2_	Solution: PMMA, MC/EtOH, ZA, HAc.	0.69	14.9	–	6.2	[39]
10	Ti-Gr	Solution: Ti(Iso), PVP, CH_3_OH, Gr. * Voltage: 12 kV Rate: 1 mL/h	0.71	16.2	0.66	7.6	[40]

**^1^** Open circuit voltage; ^2^ short circuit current density; ^3^ fill factor; ^4^ efficiency; * distance: 15 cm.

**Table 2:** Comparison of characteristics of electrospun nanofibers for counter electrodes.

Entry	Composition	Experiment Conditions	Voc ^1^ (V)	Jsc ^2^ (mA/cm^2^)	FF ^3^	η ^4^ (%)	Ref.
1	Co-TiC	Solution: PVP, HAc, EtOH, Ti(Iso), Co(CH_3_COOH)_2_ Voltage:18 kV	0.758	9.98	0.50	3.8	[42]
2	Cu_2_ZnSnS_4_	Solution: PVP, CA, EtOH, M^+2^Cl_2_, M= Cu, Zn; SnCl_4_·5H_2_O, CH_4_N_2_S. Voltage: 15 kV Rate: 1.0 mL/h	0.57	8.42	0.65	3.90	[43]
3	TiC-CNFs	Solution: TiC, PAN, DMF. * Voltage: 20 kV Rate: 0.5 mL/h	0.72	9.71	0.64	4.5	[44]
4	Ni-Co-CNFs	Solution: PAN, DMF, M^+2^(CH_3_COOH)_2_. M= Co, Ni * Voltage: 20 kV Rate: 0.5 mL/h	0.73	9.78	0.64	4.66	[45]
5	Fe-Ni-CNFs	Solution: PAN, DMF, Ni(CH_3_COOH)_2_, Fe(NO_3_)_2_ * Voltage: 25 kV Rate: 0.5 mL/h	0.72	10.1	0.65	4.7	[46]
6	Ru	Solution: PAN, DMF, RuCl_3_·xH_2_O. * Voltage: 13 kV Rate: 0.03 mL/h	0.70	14.77	0.60	6.23	[47]
7	NiCo_2_S_4_	Solution: M^+2^(NO_3_)_2_. 6H_2_O, M= Ni, Co DMF, PAN. * Voltage: 15 kV	0.70	17.06	0.60	7.12	[48]
8	Gr- CuCl_2_	Solution: PAN, CuCl_2_, InCl_3_, CH_4_N_2_S, CHCl_3_, DMF. * Voltage: 19 kV Rate: 0.2 mL/h	0.69	17.53	0.59	7.23	[49]
9	C-Pt	Solution: PAN, DMF, H_2_PtCl_6_, HCOOH Voltage: 15 kV Rate: 1.0 mL/h	0.83	13.92	0.65	7.5	[50]
10	Ni-C	Solution: PAN, DMF, Ni (AcAc)_2_ Distance: 20 cm Voltage: 18 kV	0.80	15.83	0.63	7.96	[51]

^1^ Open circuit voltage; ^2^ short circuit current density; ^3^ fill factor; ^4^ efficiency; * distance: 15 cm.

**Table 3:** Comparison of the characteristics of electrospun nanofibers for electrolytes.

Entry	Composition	Experiment Conditions	Voc ^1^ (V)	Jsc ^2^ (mA/cm^2^)	FF ^3^	η ^4^ (%)	Ref.
1	PVdF-PAN	Solution: PVDF, PAN, (CH_3_)_2_CO, DMF. * Voltage: 20 kV Rate: 1.5 mL/h	0.74	6.20	0.65	3.09	[53]
2	CA	Solution: CA, DMSO, (CH_3_)_2_CO. Distance: 10 cm Voltage: 10 kV Rate: 2 mL/h	0.699	9.83	0.58	4.0	[54]
3	SiO_2_	Solution: TEOS, PVP, HAc, EtOH. * Voltage: 20 kV Rate: 0.1 mL/min	0.60	13.63	0.59	4.85	[55]
4	PAN	Solution: PAN, DMF. Voltage: 8 kV Rate: 2 mL/h	0.67	13.31	0.59	5.3	[56]
5	PVdF-HFP	Solution: PVDF–HFP, DMF. Distance: 6.5 cm Voltage: 10 kV Rate: 1 mL/h	0.69	11.8	0.65	5.36	[57]
6	BPPO	Solution: BPPO, Et(OH), NMP. Distance: 10 cm Voltage: 16 kV Rate: 1.8 mL/min	0.70	0.58	0.58	5.4	[58]
7	PVDF–HFP/PS	Solution: PVDF–HFP, PS, DMF. * Voltage: 14 kV Rate: 2 mL/h	0.76	11.6	0.66	5.75	[59]
8	PAN-CoS	Solution: CoCl_2_·6H_2_O, L-cys, H_2_O_d_, PAN, DMF. Distance: 12 cm Voltage: 18 kV Rate: 0.5 mL/h	0.72	14.29	0.72	7.41	[60]
9	PMA-PVDF/PEG	Solution: PMA, PVDF, PGE, DMF, (CH_3_)_2_CO. * Voltage: 20 kV Rate: 0.5 mL/h	0.93	17.22	0.66	8.23	[61]
10	PVDF-LiCl	Solution: PVDF, DMF, (CH_3_)_2_CO, LiCl. * Voltage: 12 kV	0.746	14.31	0.82	8.73	[62]

^1^ Open circuit voltage; ^2^ short circuit current density; ^3^ fill factor; ^4^ efficiency; * distance: 15 cm.

## Abbreviation

**Abbreviation**	**Description**
ATN	Ag-doped TiO_2_ nanofiber
µs	Microsecond
CA	Cellulose acetate
CB	Conduction band
CE	Counter electrode
CF	Trichloromethane
CH_4_N_2_S	Thiourea
CHCl_3_	Chloroform
(CH_3_)_2_CO	Acetone
CNFs	Carbon nanofibers
Co (CH_3_COOH)_2_^.^ 4H_2_O	Cobalt acetate tetrahydrate
Co-TiC	Cobalt-titanium carbide
Cu_2_ZnSnS_4_	Sulfide kesterite
CuCl_2_	Copper (II) Chloride
DMF	Dimethylformamide
DMSO	Dimethyl Sulfoxide
DSSC	Dye sensitized solar cells
Eredox	Redox potential
EtOH	Ethanol
Fe (NO_3_)_2_	Iron(II) nitrate
FF	Fill factor
fs	Femtosecond
FTO	Fluoride doped tin oxide
GO	Graphene oxide
H_2_PtCl_6_	Chloroplatinic acid
HAc	Acetic acid
HCOOH	Formic acid
HOMO	Highest occupied molecular orbital
InCl_3_	Indium (III) Chloride
Jsc	Short circuit current density
LiCl	Lithium chloride
LUMO	Lowest unoccupied molecular orbital
M^+2^(NO_3_)_2_^.^ 6H_2_O	M= Ni, Co nickel or Cobalt nitrate hexahydrate
ms	Millisecond
η	Energy conversion efficiency
Ni (AcAc)_2_	Nickel (II) acetylacetonate
NiCo_2_S_4_	Nickel cobalt sulfide
ns	Nanosecond
NMP	N-methyl-2-pyrrolidone
PAN	Poly (acrylonitrile)
ps	Picosecond
PMMA	Poly (methyl metacrylate)
PS	Poly (styrene)
PVA	Poly (vinyl acetate)
PVDF	Poly (vinyl idene fluoride)
PVA	Poly (vinyl acetate)
PVP	Poly (vinyl pyrrolidone)
RuCl_3_·xH_2_O	Ruthenium (III) Chloride
SnCl_4_·5H_2_O	Tin(IV) Chloride Pentahydrate
TBT	Tetra-butyl titanate
TCO	Transparent conductive oxide substrate
TEOS	Tetraethyl orthosilicate
Ti-Gr	Titanium with graphene powder
Ti(Iso)	Titanium isopropoxide
TiC	Titanium carbide
Voc	Open circuit voltage
ZnCl_2_	Zinc(II) Chloride
ZA	Zirconium acetate
